# Dietary 25-hydroxycholecalciferol modulates gut microbiota and improves the growth, meat quality, and antioxidant status of growing-finishing pigs

**DOI:** 10.3389/fmicb.2022.1095509

**Published:** 2023-01-11

**Authors:** Lianhua Zhang, Shenfei Long, Hongliang Wang, Xiangshu Piao

**Affiliations:** ^1^State Key Laboratory of Animal Nutrition, College of Animal Science and Technology, China Agricultural University, Beijing, China; ^2^Key Laboratory of Plant Resources, Institute of Botany, Chinese Academy of Sciences, Beijing, China; ^3^China National Botanical Garden, Beijing, China; ^4^College of Resources and Environmental Sciences, China Agricultural University, Beijing, China

**Keywords:** 25-hydroxycholecalciferol, meat quality, antioxidant capacity, intestinal microbiota, growing-finishing pigs

## Abstract

**Introduction:**

25-Hydroxycholecalciferol (25OHD_3_) is the active metabolite of regular vitamin D_3_*in vivo*, which has a stronger biological activity and is more easily absorbed by the intestine than regular vitamin D_3_. Our study aimed to detect the potential influences of 25OHD_3_ on pork quality, antioxidant status, and intestinal microbiota of growing-finishing pigs receiving low-phosphorus (P) diet.

**Methods and results:**

Forty pigs [initial body weight (BW): 49.42 ± 4.01 kg] were allocated into two groups including low-P diet (CON group) and low-P diet supplemented with 50 μg/kg 25OHD_3_ (25OHD_3_ group). The whole experiment lasted for 88 days, including phase 1 (day 1–28), phase 2 (day 29–60), and phase 3 (day 61–88). The results showed that 25OHD_3_ supplementation tended to decrease feed conversion ratio in phase 3 and overall phase in comparison with the CON group. 25OHD_3_ increased (*p* < 0.05) serum contents of superoxide dismutase (SOD) and glutathione peroxidase (GSH-Px), and decreased (*p* < 0.05) serum bone-specific alkaline phosphatase level. 25OHD_3_ increased (*p* < 0.05) mucosal GSH-Px activity in the duodenum and ileum, and tended to increase redness value and the activities of total antioxidant capacity and SOD in *longissimus dorsi*. 25OHD_3_ significantly upregulated the mRNA level of *copper/zinc superoxide dismutase*, and tended to change the mRNA levels of *nuclear factor E2-related factor 2* and *kelch-like ECH-associated protein 1* in *longissimus dorsi*. Moreover, 25OHD_3_ supplementation decreased (*p* < 0.05) n-6/n-3 and iodine value in *longissimus dorsi*. For bone quality, 25OHD_3_ supplementation increased (*p* < 0.05) calcium content, bone mineral content, and breaking strength in the metacarpal bones. Moreover, the colonic abundance of *Lactobacillus* was significantly higher in pigs fed with 25OHD_3_, and exhibited a positive association with serum antioxidant status, pork quality, and bone characteristics.

**Conclusion:**

Overall, the inclusion of 25OHD_3_ in low P diet partly improved production performance, meat quality, antioxidant capacity, bone properties, and gut microbiota composition of growing-finishing pigs.

## Introduction

Phosphorus (P) is essential for pig production, since it is a critical component of energy transfer molecules and nucleic acids, as well as an important mineral for bone development (Lautrou et al., 2021). Sufficient P must be provided in cereal-based diets to support the growth and development of pigs. However, pigs lack endogenous phytase in the digestive tract to hydrolyze phytate, resulting in low dietary P digestibility (Zhang et al., 2021a). Therefore, farmers usually add inorganic phosphate to feed to meet the P requirements of monogastric animals. Unfortunately, inorganic phosphate is a non-renewable resource that is estimated to be depleted in 100–200 years at the current rate of exploitation (Lautrou et al., 2021). Moreover, the unreasonable use of exogenous P led to the enrichment of P in the surrounding environment, resulting in harmful algal blooms and decreased biological diversity (Zhang et al., 2021a). In turn, this issue can threaten the sustainable development of the pig industry.

Under intensive feeding conditions, bone quality of pigs is closely associated with the storage of Ca and P, and is influenced by dietary Ca and P levels. Ca and P deficiency can impair bone development and peak bone mass. Leg weakness is a critical issue for fast-growing pigs, which can further compromise the production performance of fattening pigs (Jørgensen, 1995; Sørensen et al., 2018). It has been reported that lower Ca level decreased the antioxidant enzyme activity including superoxide dismutase (SOD; Choi and Jung, 2017), while insufficient P reduced glutathione content and suppressed the activities and mRNA expressions of the antioxidant enzymes (Chen et al., 2017). It suggested that dietary Ca and P deficiency can promote oxidative damage in pigs. Moreover, dietary P deficiency can impair meat quality in broilers, which led to lower intramuscular fat (IMF) and fatty acids in breast meat (Li et al., 2016). The fatty acid profile in the IMF of muscle is related to meat quality and plays an essential role in pork taste and flavor (Moeller et al., 2010). It has been demonstrated that P is important for maintaining immune function in the intestine (Heyer et al., 2015), and sufficient Ca-P in diets downregulated the mRNA expression of interleukin-1β in the duodenum in comparison with pigs under dietary Ca and P deficiency (Metzler-Zebeli et al., 2012). It suggested that dietary P deficiency may hamper intestinal health. In this regard, how to maintain the performance and health of pigs fed with low P is a problem worthy of further study.

Vitamin D_3_ can regulate intestinal absorption of Ca and P, and maintain optimal homeostasis for bone mineralization (Haussler et al., 2013; Dal Jang et al., 2018). In addition to this function, vitamin D_3_ can improve the antioxidant status and immunity of pigs, promote the differentiation of the intestinal epithelial cells, and regulate the gut microbiota community (Tian et al., 2016; Rey et al., 2020; Zhang and Piao, 2021; Zhang et al., 2022b). Vitamin D_3_ plays an important role in promoting antioxidant ability in the loin muscle of pigs, which contributes to extending shelf life and supporting meat quality (Zhang et al., 2021b). Importantly, it can also enhance dietary phytate *P* availability by promoting phytase activity in the intestine (Zhang et al., 2022c). However, the effectiveness of regular vitamin D_3_ is low, which is difficult to meet the requirements of rapid growth of pigs. The bioavailability of 25-hydroxycholecalciferol (25OHD_3_) is higher than vitamin D_3_ (Chou et al., 2009), and it is biologically safer than 1,25-dihydroxycholecalciferol (Soares Jr. et al., 1995). However, little information is available on the potential influences of 25OHD_3_ on pork quality, antioxidant ability, and intestinal function of pigs receiving low dietary P supplementation.

Given this background, we hypothesize that 25OHD_3_ can improve production performance, meat quality, antioxidant capacity, and intestinal health of growing-finishing pigs fed with low P.

## Materials and methods

### Animals, diets and experimental design

A total of 40 Duroc × Landrace × Yorkshire crossbred pigs [initial body weight (BW): 49.42 ± 4.01 kg] were randomly assigned to two groups, and each group had five replicates. The two dietary treatments were as follows: (1) low P diet (CON); (2) low P diet +50 μg/kg 25OHD_3_ (25OHD_3_). Feed-grade 25OHD_3_ (0.125%) was supported by Haineng Bioengineering Co., Ltd., (Rizhao, China). The whole experiment was divided into three phases: (1) day 1–28, (2) day 29–60, and (3) day 61–88. We estimated the duration of each phase of pigs based on the NRC (2012). The Ca-P levels in the experimental diets (Table 1) were 0.43% total P, 0.19% available P and 0.52% Ca for phase 1, 0.39% total P, 0.17% available P and 0.46% Ca for phase 2, 0.35% total P, 0.15% available P and 0.42% Ca for phase 3, respectively. Except for Ca and P, all other nutrients met the recommended requirements. Dietary samples were detected based on the analysis methods of AOAC (2006) for Ca (method 968.08), P (method 985.01), and crude protein (method 990.03). All pigs had free access to mash feed and fresh water for 88 day. The amounts of feed intake and residual feed for pigs were recorded daily on a pen basis to calculate average daily feed intake (ADFI) and all pigs were weighed after fasting on day 0, 28, 60, and 88 of this experiment to calculate the average daily gain (ADG), then feed conversion ratio was calculated (FCR = ADFI/ADG).

**Table 1:** Composition and nutrient levels of the basal diets (%, as-fed basis).

Item	Phase 1 (50–75 kg)	Phase 2 (75–100 kg)	Phase 3 (100–slaughter)
Ingredients			
Corn	69.57	75.24	80.40
Soybean meal	20.00	15.00	10.50
Wheat bran	8.00	7.50	7.00
Dicalcium phosphate	0.18	0.08	-
Limestone	1.10	1.05	1.00
Salt	0.30	0.30	0.30
L-Lysine HCl	0.25	0.24	0.22
DL-Methionine	0.03	0.02	0.01
L-Threonine	0.05	0.05	0.05
L-Tryptophan	0.02	0.02	0.02
Vitamin and mineral premix^1^	0.50	0.50	0.50
Analyzed nutrient levels			
CP	15.50	13.70	12.10
Ca	0.52	0.46	0.42
Total *P*	0.43	0.39	0.35
Calculated nutrient levels			
Digestible energy (Mcal/kg)	3.40	3.40	3.40
Available *P*	0.19	0.17	0.15
SID Lys	0.86	0.73	0.62
SID Met	0.24	0.22	0.19
SID Thr	0.53	0.47	0.41
SID Try	0.16	0.14	0.11

^1^Premix used in phase 1: Vitamin A, 5,600 IU; Vitamin D_3_, 2,200 IU; Vitamin E, 21.6 IU; Vitamin K_3_, 1.8 mg; Vitamin B_1_, 0.88 mg; Vitamin B_2_, 4 mg; Vitamin B_6_, 1.8 mg; Vitamin B_12_, 12 μg; Pantothenic acid, 10 mg; Nicotinic acid, 20 mg; Folic acid, 0.4 mg; Choline chloride, 0.32 g; Biotin, 40 μg; Cu, 15 mg; Fe, 100 mg; I, 0.3 mg; Mn, 10 mg; Se, 0.3 mg. Phase 2 and phase 3: Vitamin A, 5,200 IU; Vitamin D_3_, 2,000 IU; Vitamin E, 17.2 IU; Vitamin K_3_, 1.6 mg; Vitamin B_1_, 0.8 mg; Vitamin B_2_, 3.6 mg; Vitamin B_6_, 1.6 mg; Vitamin B_12_, 10 μg; Pantothenic acid, 8.8 mg; Nicotinic acid, 17.6 mg; Folic acid, 0.38 mg; Choline chloride, 0.24 g; Biotin, 32 μg; Cu, 15 mg; Fe, 80 mg; I, 0.2 mg; Mn, 5 mg; Se, 0.3 mg.

### Sample collection

On day 28, 60, and 88 of this experiment, one barrow close to the average BW of each pen was selected. Blood (10 ml) was collected *via* the jugular vein, and the sample was centrifuged at 3000 × *g* for 10 min at 4°C after standing for 3 h, then collected the serum and stored at −20°C for analysis. On day 88, one barrow close to the average BW of each pen was humanly slaughtered after electrical stunning (Zhang et al., 2021b). The collecting methods of tissue samples refer to Zhang et al. (2021a,b). Immediately after death, mucosal samples were scraped from the middle parts of the duodenum, jejunum and ileum and stored at −80°C for analysis of antioxidant parameters. Colonic contents were transferred to sterile tubes and stored at −80°C for analysis of gut microbiota. The *longissimus dorsi* from the left sides of the carcasses was collected between the 10th and 11th ribs for analysis of meat quality and chemical composition. The third and fourth metacarpal bones of the front feet were collected and then frozen at −20°C.

### Serum parameters

According to the method of Zhang et al. (2022b), the serum contents of total antioxidant capacity (T-AOC, A015-3-1), superoxide dismutase (SOD, A001-3), catalase (CAT, A007-1-1), glutathione peroxidase (GSH-Px, A005-1), and malondialdehyde (MDA, A003-1) were measured using colorimetric kits (Nanjing Jiancheng Bioengineering Institute, Jiangsu, China). According to the method of Zhang et al. (2019), serum concentrations of bone-specific alkaline phosphatase (BALP, HY-10285), tartrate-resistant acid phosphatase (TRAP, HY-10289), osteocalcin (OC, HY-10160), and parathyroid hormone (PTH, HY-10178) were determined using commercial kits (Sino-United Kingdom Institute of Biological Technology, Beijing, China).

### Carcass traits and meat quality

Hot carcass weight was determined immediately, and the dressing percentage was obtained by dividing the hot carcass weight by the live weight. Backfat thickness was determined using a vernier caliper at the midline of the 10th rib. Carcass oblique length was recorded as the distance from the first rib to the aitch bone. The longissimus muscle area was calculated according to the formula: longissimus muscle area (cm^2^) = height (cm) × width (cm) × 0.7. The values of pH_45 min_ and pH_24 h_ were measured using a SPK pH meter (pH-star, DK2730, Herlev, Denmark). At 45 min postmortem, meat color including lightness (L*), redness (a*), and yellowness (b*) was measured using a colorimeter (CR-410, MINOLTA, Japan) according to CIE lab color system. Additionally, chroma (C*) and hue angle (*h*°) were calculated by the formula: C* = (a*^2^ + b*^2^)^0.5^ and *h*° = arctg b*/a*. Drip loss (%) was calculated as fluid loss after suspending muscle samples in plastic bags at 4°C for 24 h. The contents of CP, IMF, Ca, and P in muscle samples were analyzed according to the corresponding methods of AOAC (2006).

### Antioxidant parameters in the small intestinal mucosa and *longissimus dorsi*

The frozen samples of the intestinal mucosa and *longissimus dorsi* were homogenized in ice-cold saline solution (1:9, wt/vol) with an Ultra-Turrax homogenizer (Bioblock Scientific, Illkirch, France). The homogenate was centrifuged at 2500 × *g* for 10 min at 4°C, and the supernatant was analyzed for antioxidant parameters. The contents of T-AOC, SOD, CAT, GSH-Px, MDA, protein carbonyl (PC), and 8-hydroxy-2′-deoxyguanosine (8-OHdG) in the intestinal mucosa and *longissimus dorsi* were analyzed using commercial kits (Nanjing Jiancheng Bioengineering Institute, Jiangsu, China).

### Muscle fatty acid profile

Lyophilized Muscle samples (200 mg) were mixed with 4 ml acetyl chloride/methanol [(v/v), 1:10] solution, 1 ml n-hexane, and 1 ml internal standard, and then kept in a water bath at 80°C for 2.5 h. After cooling, 5 ml of 7% potassium carbonate solution was added and then centrifuged at 150 × *g* for 5 min. The muscle fatty acid profile was determined using a gas chromatograph (6,890 series, Agilent Technologies, Wilmington, DE) equipped with a DB-23 capillary column (length 60 m, internal diameter 0.25 mm, film thickness 0.25 μm; Agilent Technologies Inc., Santa Clara, CA, United States). Fatty acid profile was expressed as the proportion of each individual fatty acid to the total of all fatty acids present in the sample.

### RNA extraction and qRT-PCR analysis

According to the method of Zhang et al. (2021b), total RNA was extracted from *longissimus dorsi* using Trizol reagent (CWBIO Biotech Co., Beijing, China) and then reverse-transcribed into cDNA using the PrimeScript^RT^ Reagent Kit (TaKaRa, Dalian, China). SYBR Green-based RT-PCR was performed using the ABI Prism 7,500 Sequence Detection System (Applied Biosystems). The relative mRNA expression relative to a housekeeping gene (*GAPDH*) was calculated using the 2^−ΔΔCT^ method (Xu et al., 2022). Primer sequences were listed in Table 2.

**Table 2:** Primer sequences for real-time PCR analysis.

Gene	Primer sequence (5′-3′)	Size (bp)	GenBank accession No.
*Cu/Zn SOD*	F: AACCAGATGACTTGGGCAGA	120	NM_001190422.1
	R: AGACCATGGCATGAGGGAAT		
*CAT*	F: AGATGGACACAGGCACATGA	111	NM_214301.2
	R: TTGATGCCCTGGTCAGTCTT		
*GPx1*	F: GGTTCGAGCCCAACTTCATG	165	NM_214201.1
	R: CATTGCGACACACTGGAGAC		
*GPx4*	F: TGTGGTTTACGGATTCTGG	181	NM_214407.1
	R: CCTTGGGCTGGACTTTCA		
*Nrf2*	F: AGAGCCCAGTCTTCATTGCT	96	XM_021075132.1
	R: TGTCCTGTTGCATACCGTCT		
*Keap1*	F: TACATGCACTTTGGGGAGGT	89	XM_005654811.3
	R: AGATCGTCCCGGCTAATGAG		
*GAPDH*	F: TCGGAGTGAACGGATTTGGC	189	NM_001206359.1
	R: TGACAAGCTTCCCGTTCTCC		

Cu/Zn SOD, copper/zinc superoxide dismutase; CAT, catalase; GPx1, glutathione peroxidase 1; GPx4, glutathione peroxidase 4; Nrf2, nuclear factor E2-related factor 2; Keap1, kelch-like ECH-associated protein 1; GAPDH, glyceraldehyde-3-phosphate dehydrogenase.

### Bone analysis

Bone mineral content (BMC) and bone mineral density (BMD) were measured using a Medilink Bone Densitometer (Model Lexxos-2000, MEDILINK, Montpellier, France) *via* the dual-energy X-ray absorptiometry method. The bone mechanical property was measured using MTS Material Testing Apparatus (Model 810, MTS Systems Corporation, Minneapolis, United States) by a three-point bending test. Briefly, the load was applied to the midpoint of the bone samples, which was held by two supports spaced 30 mm apart. The load–displacement curves were recorded at a compression speed of 10 mm/min, and breaking strength, failure deflection, stiffness, and absorbed energy were determined. The contents of Ash, Ca, and P were analyzed according to the methods described by Zhang et al. (2019).

### Colonic microbiota analysis

According to the method of Zhang et al. (2022c), genomic DNA of colonic contents was extracted using the DNA Kit (Omega Bio-Tek, Norcross, GA, United States). The V3-V4 regions of the microbial 16S rRNA gene were amplified using primers 338F (5’-ACTCCTRCGGGAGGCAGCAG-3′) and 806R (5’-GGACTACCVGGGTATCTAAT-3′). The PCR products were extracted using 2% agarose gel and then purified. The purified amplicons were pooled and paired-end sequenced *via* the Illumina MiSeq platform (Illumina Inc., San Diego, CA, USA). UPARSE software (v 7.1^1^) was used to cluster the remaining high-quality sequences into operational taxonomic units with a similarity of 97%. The RDP Classifier (v 2.13^2^) was used to determine the taxonomy of each 16S rRNA gene sequence with confidence greater than 70%. Data analysis was conducted by the Majorbio Cloud Platform^3^.

### Microbial metabolites

The concentrations of short-chain fatty acids (SCFAs) was determined using an ion chromatography system (DIONEX ICS-3000, Thermo Fisher, Waltham, MA, United States). Colonic contents (0.5 g) were mixed with 8 ml ultrapure water, homogenized and then centrifuged at 3,000 × *g* for 5 min. The supernatants were diluted 50 times and filtered through a 0.22 μm membrane before injection into an AG11 guard column (250 mm × 4 mm) and an AG11 guard column using KOH for isocratic elution. The injection volume was 25 μl and the flow rate was 1.0 ml/min. The contents of SCFAs were expressed as mg/g of the digesta.

### Statistical analysis

Data analysis was conducted using SAS 9.4 (SAS Inst. Inc., Cary, NC). The Shapiro–Wilk and Levene’s tests were used to verify the normal distribution and homogeneity of variances of the data. The independent sample *t-*test procedure was used to detect significant differences between the two groups. Serum parameters were analyzed using two-factor repeated measurements of SAS (Treatments with collection time point as the repeated measurement). The Mann–Whitney U test was used to analyze the relative abundance of bacteria at the taxonomic levels of phylum, family, and genus. Correlations between the relative abundance of *Lactobacillus* in the colonic digesta and serum parameters, pork quality parameters, bone parameters or colonic metabolites were tested by Spearman’s correlation analysis of the SAS system. All data were shown as the means ± SEM. *p* < 0.05 was considered a significant difference, and 0.05 ≤ *p* < 0.10 was considered a significant trend.

## Results

### Performance and carcass traits

The growth performance is shown in Table 3. 25OHD_3_ tended to reduce the FCR of pigs during day 61–88 (*p* = 0.08) and day 1–88 (*p* = 0.09). However, ADG and ADFI were not affected by 25OHD_3_ supplementation. In addition, 25OHD_3_ did not change carcass traits (Table 4).

**Table 3:** Effects of 25OHD_3_ supplementation on growth performance of growing-finishing pigs.

Items	CON	25OHD_3_	*p*-value
Initial BW (kg)	49.35 ± 2.15	49.49 ± 2.18	0.96
d 28 BW (kg)	70.99 ± 3.45	72.19 ± 2.17	0.78
d 60 BW (kg)	95.14 ± 5.43	98.26 ± 5.20	0.69
d 88 BW (kg)	117.40 ± 6.03	122.94 ± 6.55	0.56
d 1–28			
ADG (kg)	0.77 ± 0.05	0.82 ± 0.01	0.38
ADFI (kg)	2.22 ± 0.20	2.00 ± 0.05	0.26
FCR	2.91 ± 0.32	2.44 ± 0.07	0.24
d 29–60			
ADG (kg)	0.75 ± 0.08	0.82 ± 0.11	0.65
ADFI (kg)	2.45 ± 0.19	2.52 ± 0.24	0.83
FCR	3.30 ± 0.17	3.14 ± 0.20	0.57
d 61–88			
ADG (kg)	0.80 ± 0.03	0.89 ± 0.04	0.15
ADFI (kg)	2.74 ± 0.16	2.72 ± 0.21	0.85
FCR	3.42 ± 0.13	3.05 ± 0.12	0.08
d 1–88			
ADG (kg)	0.77 ± 0.05	0.83 ± 0.05	0.43
ADFI (kg)	2.47 ± 0.12	2.42 ± 0.17	0.81
FCR	3.21 ± 0.12	2.90 ± 0.09	0.09

Values are shown as the means ± SEM, *n* = 5. BW, body weight; ADG, average daily gain; ADFI, average daily feed intake; FCR, feed conversion ratio.

**Table 4:** Effects of 25OHD_3_ supplementation on carcass traits of growing-finishing pigs.

Items	CON	25OHD_3_	*p*-value
Hot carcass weight (kg)	91.45 ± 1.22	92.02 ± 1.49	0.78
Dressing percentage (%)	71.96 ± 0.71	72.40 ± 0.49	0.62
Carcass oblique length (cm)	91.80 ± 0.51	92.00 ± 1.00	0.86
Back fat thickness (mm)	20.68 ± 1.18	22.52 ± 0.56	0.20
Longissimus muscle area (cm^2^)	61.16 ± 1.86	62.47 ± 0.61	0.52

Values are shown as the means ± SEM, *n* = 5.

### Serum parameters

**Figure 1:**
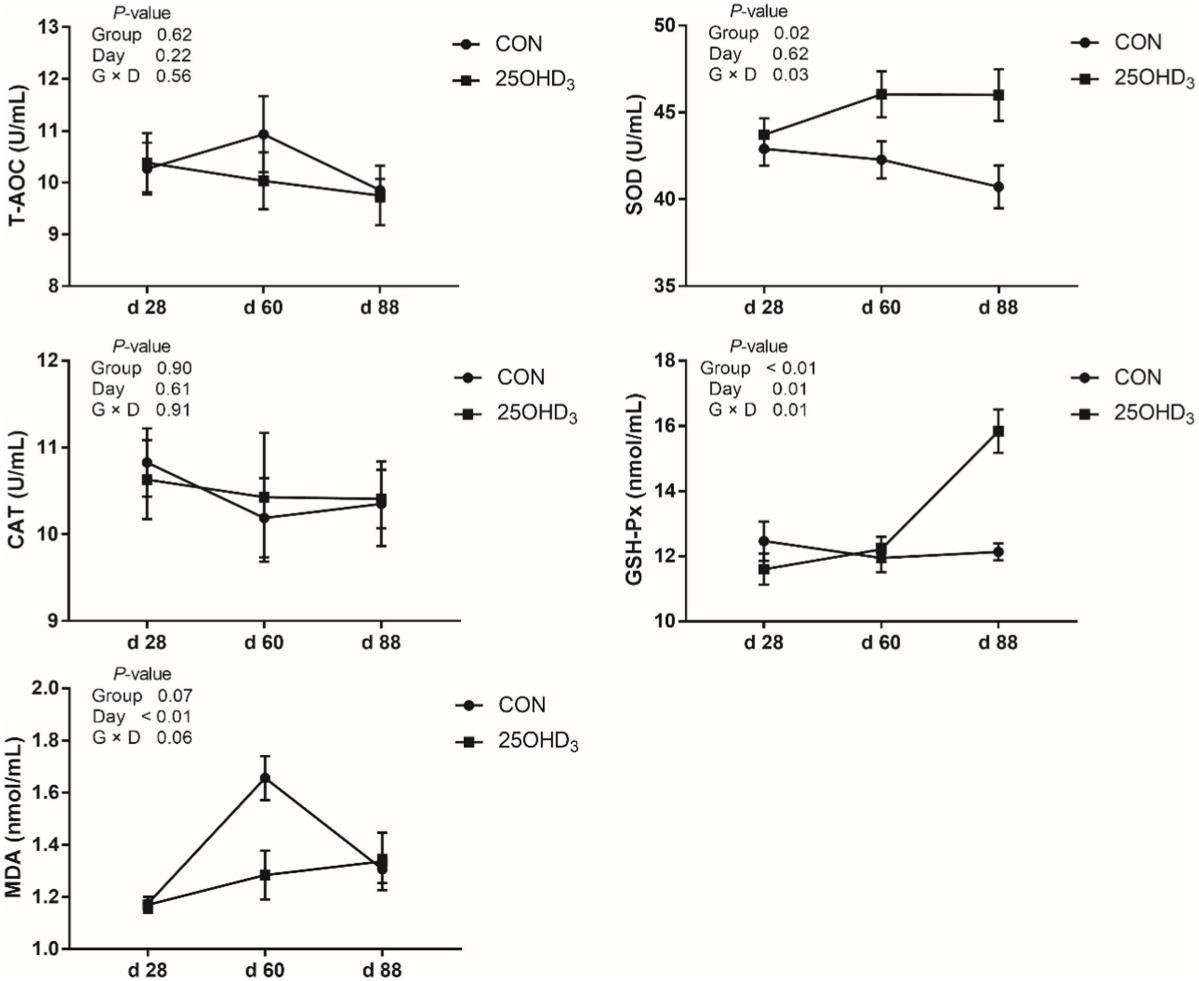
The serum antioxidant parameters between the two treatments. Values are shown as the means ± SEM, *n* = 5. T-AOC, total antioxidant capacity; SOD, superoxide dismutase; CAT, catalase; GSH-Px, glutathione peroxidase; MDA, malondialdehyde.

**Figure 2:**
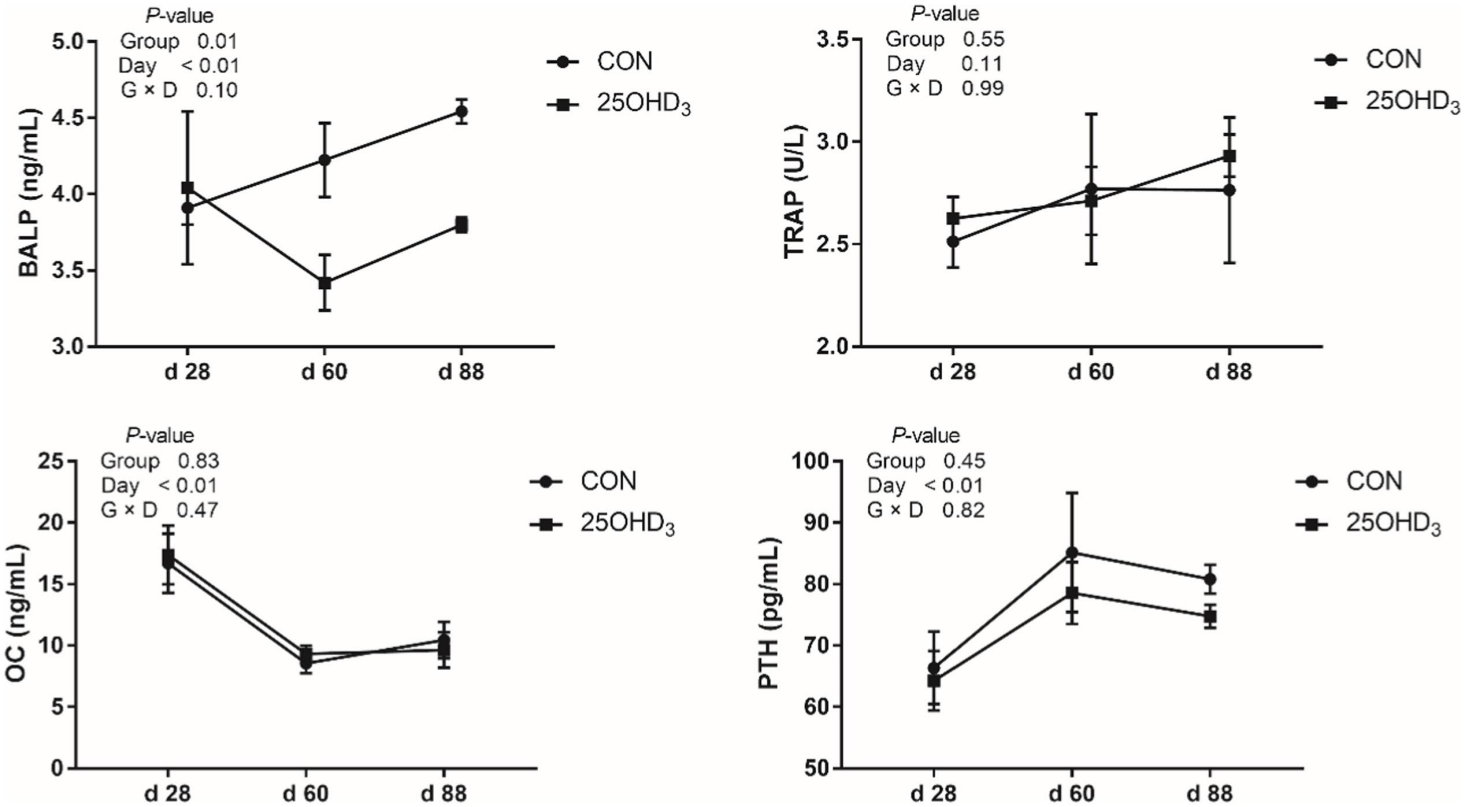
The serum bone biochemical markers between the two treatments. Values are shown as the means ± SEM, *n* = 5. BALP, bone-specific alkaline phosphatase; TRAP, tartrate-resistant acid phosphatase; OC, osteocalcin; PTH, parathyroid hormone.

As shown in Figure 1, 25OHD_3_ increased (*p* < 0.05) serum contents of SOD and GSH-Px, and tended to decrease serum MDA level. Moreover, 25OHD_3_ decreased (*p* < 0.05) serum BALP content (Figure 2).

### Meat quality and chemical composition

As shown in Table 5, 25OHD_3_ supplementation tended to increase (*p* = 0.07) muscle a* value, but did not change other parameters of meat quality. Moreover, 25OHD_3_ enhanced (*p* < 0.05) muscle Ca content.

**Table 5:** Effects of 25OHD_3_ supplementation on meat quality and chemical composition of *longissimus dorsi*.

Items	CON	25OHD_3_	*p*-value
pH_45 min_	6.32 ± 0.16	6.29 ± 0.06	0.89
pH_24 h_	5.43 ± 0.04	5.48 ± 0.02	0.36
Drip loss (%)	7.44 ± 1.08	7.78 ± 0.98	0.82
Color			
L* (lightness)	52.07 ± 0.41	52.74 ± 0.37	0.26
a* (redness)	5.93 ± 0.11	6.15 ± 0.05	0.07
b* (yellowness)	3.93 ± 0.08	3.95 ± 0.07	0.83
Chroma	7.12 ± 0.08	7.25 ± 0.05	0.20
Hue (degrees)	1920.93 ± 54.41	1908.98 ± 24.49	0.85
Chemical composition			
Crude protein (g/100 g)	22.46 ± 0.66	22.62 ± 0.76	0.88
Intramuscular fat (g/100 g)	1.79 ± 0.15	1.89 ± 0.16	0.65
Calcium (mmol/g)	0.19 ± 0.01	0.22 ± 0.01	0.02
Phosphorus (mmol/g)	0.50 ± 0.03	0.51 ± 0.01	0.68

Values are shown as the means ± SEM, *n* = 5.

### Muscle fatty acid profile

The muscle fatty acid profile is shown in Table 6. 25OHD_3_ supplementation significantly increased muscle C14:0 content and decreased n-6/n-3PUFA and iodine value. The percentages of C12:0 and SFA tended to be increased in *longissimus dorsi* of pigs.

**Table 6:** Effects of 25OHD_3_ supplementation on the fatty acid profile of *longissimus dorsi* (g/100 g total fatty acid).

Items	CON	25OHD_3_	*p*-value
C10:0 (Decanoic acid)	0.20 ± 0.01	0.22 ± 0.01	0.15
C12:0 (Lauric acid)	0.10 ± 0.002	0.11 ± 0.002	0.08
C14:0 (Myristic acid)	1.49 ± 0.02	1.58 ± 0.01	<0.01
C16:0 (Palmitic acid)	26.70 ± 0.37	27.05 ± 0.22	0.44
C16:1 (Palmitoleic acid)	3.20 ± 0.06	3.15 ± 0.18	0.81
C17:0 (Margaric acid)	0.15 ± 0.01	0.16 ± 0.01	0.58
C18:0 (Stearic acid)	14.75 ± 0.23	15.58 ± 0.59	0.23
C18:1n-9 (Oleic acid)	41.97 ± 0.82	41.38 ± 0.63	0.58
C18:2n-6 (Linoleic acid)	7.65 ± 0.54	6.85 ± 0.24	0.21
C18:3n-3 (Linolenic acid)	0.21 ± 0.01	0.21 ± 0.02	0.92
C20:0 (Arachidic acid)	0.30 ± 0.01	0.29 ± 0.01	0.37
C20:1 (Eicosenoic acid)	0.75 ± 0.04	0.64 ± 0.04	0.11
C21:0 (Heneicosanoic acid)	0.32 ± 0.01	0.34 ± 0.03	0.64
C20:3n-6 (Carbonium acid)	0.18 ± 0.02	0.22 ± 0.02	0.31
C20:4n-6 (Arachidonic acid)	1.31 ± 0.29	1.65 ± 0.21	0.44
SFA	43.94 ± 0.58	45.39 ± 0.48	0.09
MUFA	46.18 ± 0.83	45.29 ± 0.80	0.46
PUFA	9.47 ± 0.80	9.11 ± 0.46	0.71
n-6 PUFA	9.14 ± 0.78	8.72 ± 0.43	0.65
n-3 PUFA	0.33 ± 0.02	0.39 ± 0.04	0.24
n-6/n-3	27.27 ± 0.84	22.77 ± 1.34	0.02
Iodine value	53.55 ± 0.41	51.53 ± 0.50	0.01

Values are shown as the means ± SEM, *n* = 5. SFA, saturated fatty acid; MUFA, monounsaturated fatty acid; PUFA, polyunsaturated fatty acid. Iodine value = (0.95 × C16:1) + (0.86 × C18:1) + (1.732 × C18:2) + (2.616 × C18:3) + (0.785 × C20:1) + (0.723 × C22:1) (Zhang et al., 2021b).

### Antioxidant status of the small intestinal mucosa and *longissimus dorsi*

**Figure 3:**
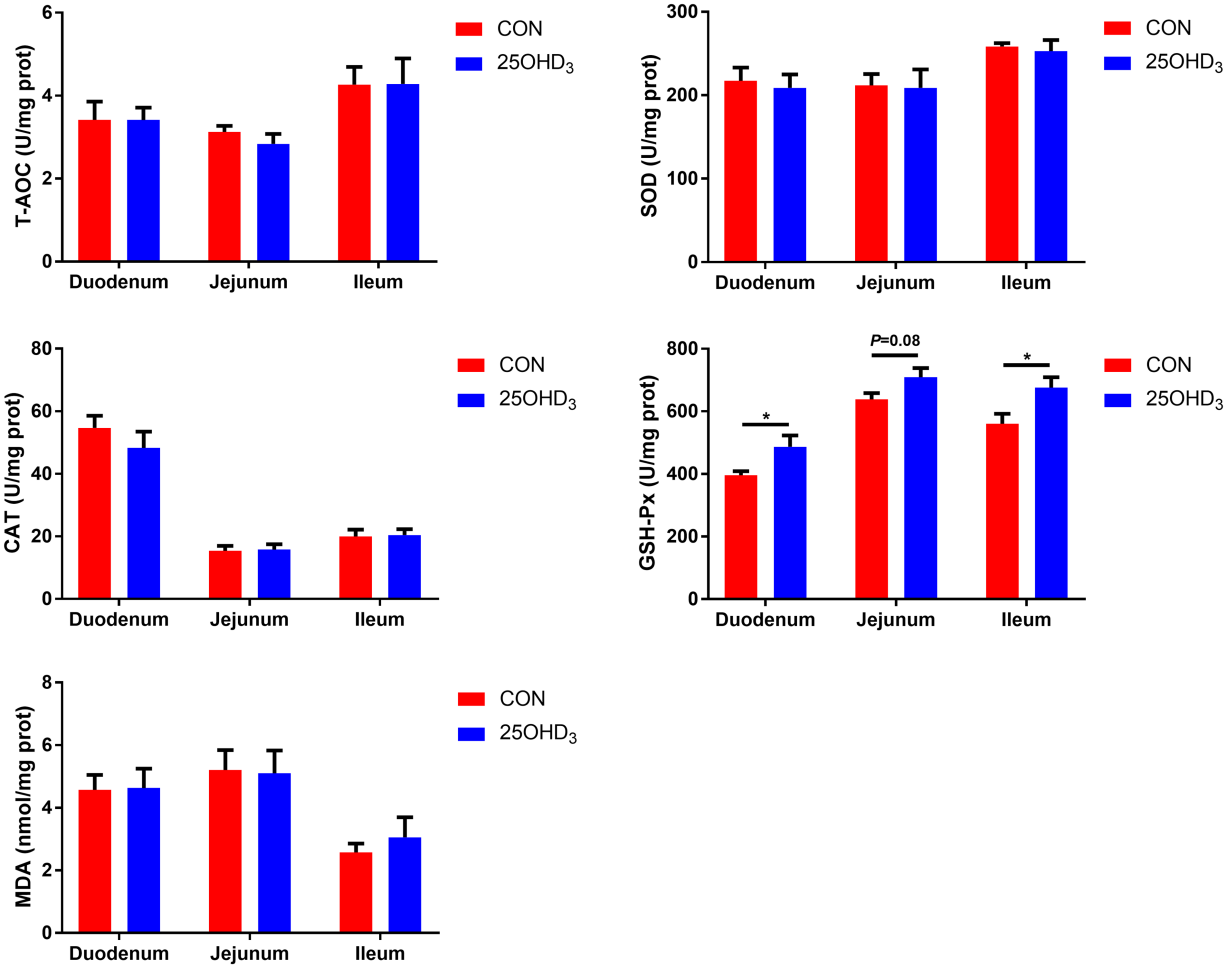
The mucosal antioxidant parameters between the two treatments. Values are shown as the means ± SEM, *n* = 5. *p < 0.05 versus CON group. T-AOC, total antioxidant capacity; SOD, superoxide dismutase; CAT, catalase; GSH-Px, glutathione peroxidase; MDA, malondialdehyde.

**Figure 4:**
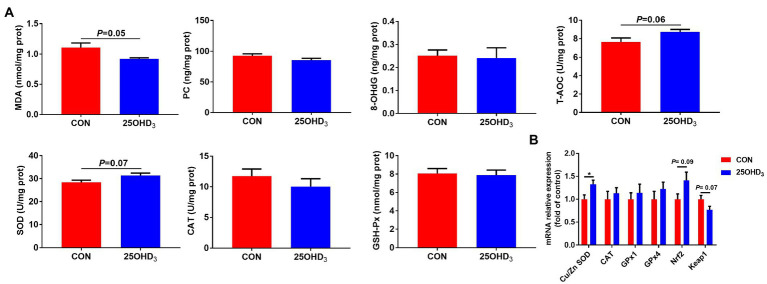
The antioxidant status and mRNA expression of antioxidant-related genes between the two treatments. **(A)** Antioxidant status in *longissimus dorsi*. **(B)** The mRNA expression of antioxidant-related genes in *longissimus dorsi*. Values are shown as the means ± SEM, *n* = 5. *p < 0.05 versus CON group. MDA, malonaldehyde; PC, protein carbonyl; 8-OHdG, 8-hydroxy-2′-deoxyguanosine; T-AOC, total antioxidant capacity; SOD, superoxide dismutase; CAT, catalase; GSH-Px, glutathione peroxidase; Cu/Zn SOD, copper/zinc superoxide dismutase; GPx1, glutathione peroxidase 1; GPx4, glutathione peroxidase 4; Nrf2, nuclear factor E2-related factor 2; Keap1, kelch-like ECH-associated protein 1.

As shown in Figure 3, 25OHD_3_ supplementation increased (*p* < 0.05) mucosal GSH-Px content in the duodenum and ileum, and tended to increase (*p* = 0.08) GSH-Px activity in the jejunal mucosa. Dietary 25OHD_3_ supplementation tended to reduce the muscle content of MDA and increase the muscle contents of T-AOC and SOD (Figure 4A). 25OHD_3_ upregulated (*p* < 0.05) copper/zinc superoxide dismutase (*Cu/Zn SOD*) mRNA level, and tended to upregulate (*p* = 0.09) nuclear factor E2-related factor 2 (*Nrf2*) mRNA level and downregulate (*p* = 0.07) kelch-like ECH-associated protein 1 (*Keap1*) mRNA level in *longissimus dorsi* (Figure 4B).

### Bone quality

As shown in Table 7, 25OHD_3_ supplementation significantly enhanced Ca content, BMC and breaking strength, as well as tended to enhance P content and stiffness in the metacarpal bones.

**Table 7:** Effects of 25OHD_3_ supplementation on bone property in the metacarpal bones of growing-finishing pigs.

Item	CON	25OHD_3_	*p*-value
Third metacarpal bone			
Bone mineral content (g)	3.06 ± 0.09	3.54 ± 0.11	0.01
Bone mineral density (g/cm^2^)	0.34 ± 0.01	0.32 ± 0.02	0.41
Ash (%)	42.70 ± 0.55	43.61 ± 0.64	0.31
Calcium (%)	16.01 ± 0.07	16.56 ± 0.13	<0.01
Phosphorus (%)	7.25 ± 0.09	7.50 ± 0.08	0.07
Breaking strength (N)	1387.24 ± 36.68	1508.42 ± 34.52	0.04
Failure deflection (mm)	3.99 ± 0.40	3.79 ± 0.30	0.69
Stiffness (N/mm)	531.02 ± 35.88	618.82 ± 23.34	0.07
Absorbed energy (J)	4.01 ± 0.56	3.86 ± 0.20	0.80
Fourth metacarpal bone			
Bone mineral content (g)	2.92 ± 0.10	3.28 ± 0.12	0.04
Bone mineral density (g/cm^2^)	0.31 ± 0.01	0.30 ± 0.01	0.66
Ash (%)	42.55 ± 0.60	42.17 ± 0.43	0.62
Calcium (%)	16.00 ± 0.06	16.44 ± 0.10	<0.01
Phosphorus (%)	7.19 ± 0.06	7.36 ± 0.07	0.08
Breaking strength (N)	1289.12 ± 34.49	1430.59 ± 37.20	0.02
Failure deflection (mm)	4.00 ± 0.10	4.03 ± 0.30	0.92
Stiffness (N/mm)	525.98 ± 23.25	590.50 ± 17.70	0.06
Absorbed energy (J)	4.12 ± 0.31	3.99 ± 0.23	0.76

Values are shown as the means ± SEM, *n* = 5.

### Colonic microbiota

**Figure 5:**
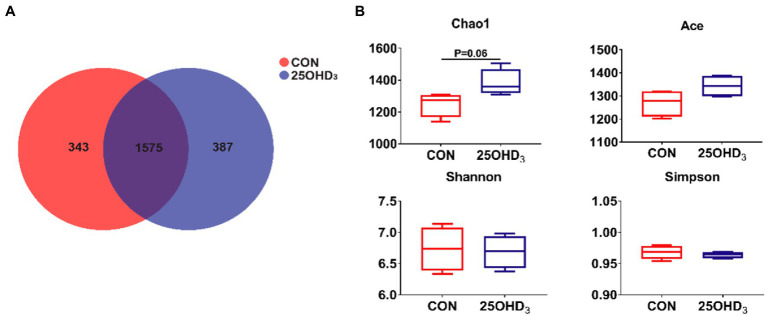
The colonic microbiota richness and diversity. **(A)** OTU Venn. **(B)** Comparison of a-diversity indices between the two treatments. Values are shown as the means ± SEM, *n* = 5.

The richness and diversity of colonic microbiota are shown in Figure 5. The Venn analysis identified 343 and 387 unique OTU in the CON and 25OHD_3_ groups, respectively, of which 1,575 were shared OTUs between the two treatments (Figure 5A). 25OHD_3_ tended to increase (*p* = 0.06) chao 1 index in the colonic digesta compared with control (Figure 5B).

**Figure 6:**
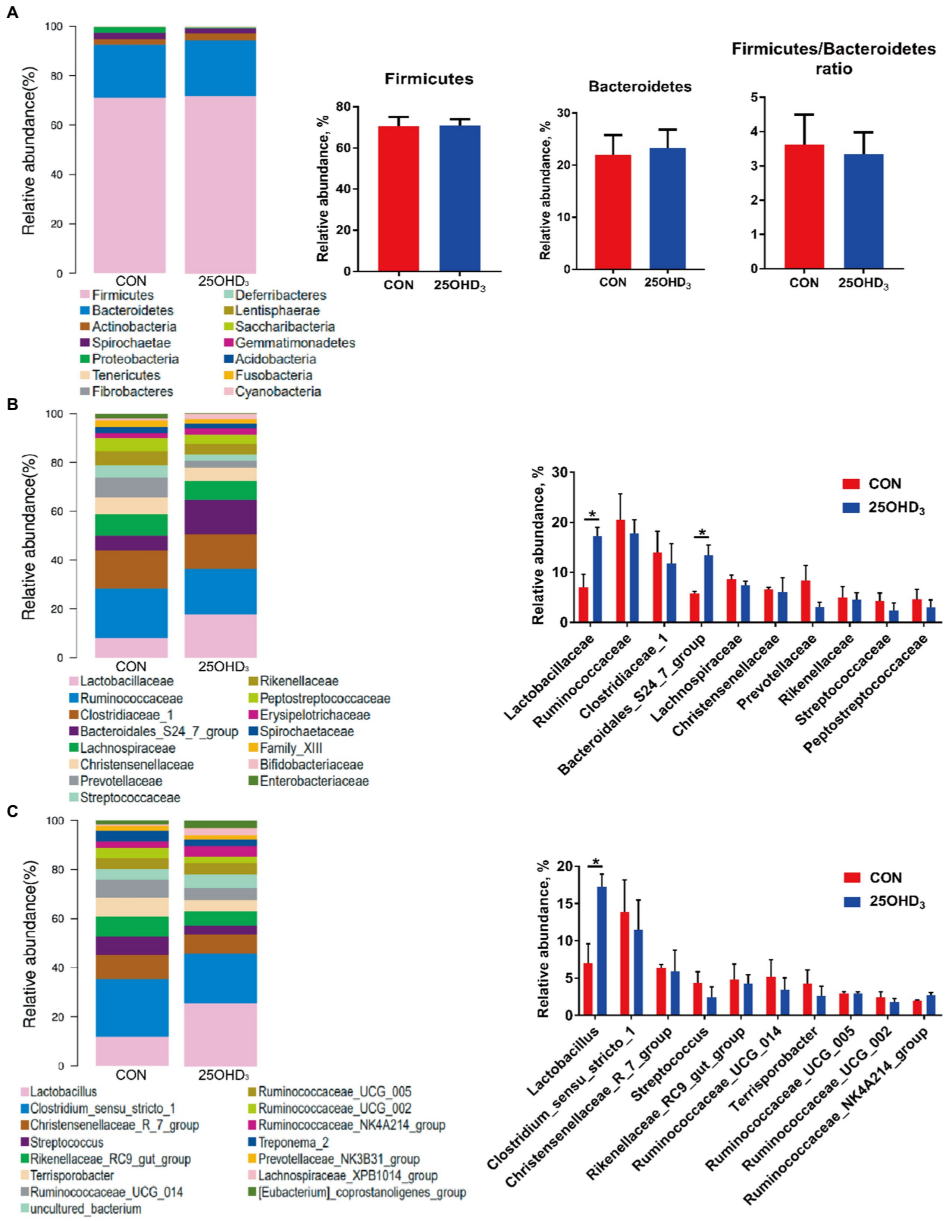
The composition of colonic microbiota at the phylum **(A)**, family **(B)** and genus **(C)** levels. Values are shown as the means ± SEM, *n* = 5. *p < 0.05 versus CON group.

The composition of colonic microbiota is shown in Figure 6. The dominant bacteria were Firmicutes and Bacteroidetes, accounting for more than 90% (Figure 6A). No differences were observed for the abundances of Firmicutes, Bacteroidetes, and their ratio. The relative abundances of colonic Lactobacillaceae and Bacteroidales_S24_7_group in the 25OHD_3_ group were higher (*p* < 0.05) than control (Figure 6B). The abundance of colonic *Lactobacillus* in the 25OHD_3_ group was higher (*p* < 0.05) than control (Figure 6C).

### Colonic metabolites

**Figure 7:**
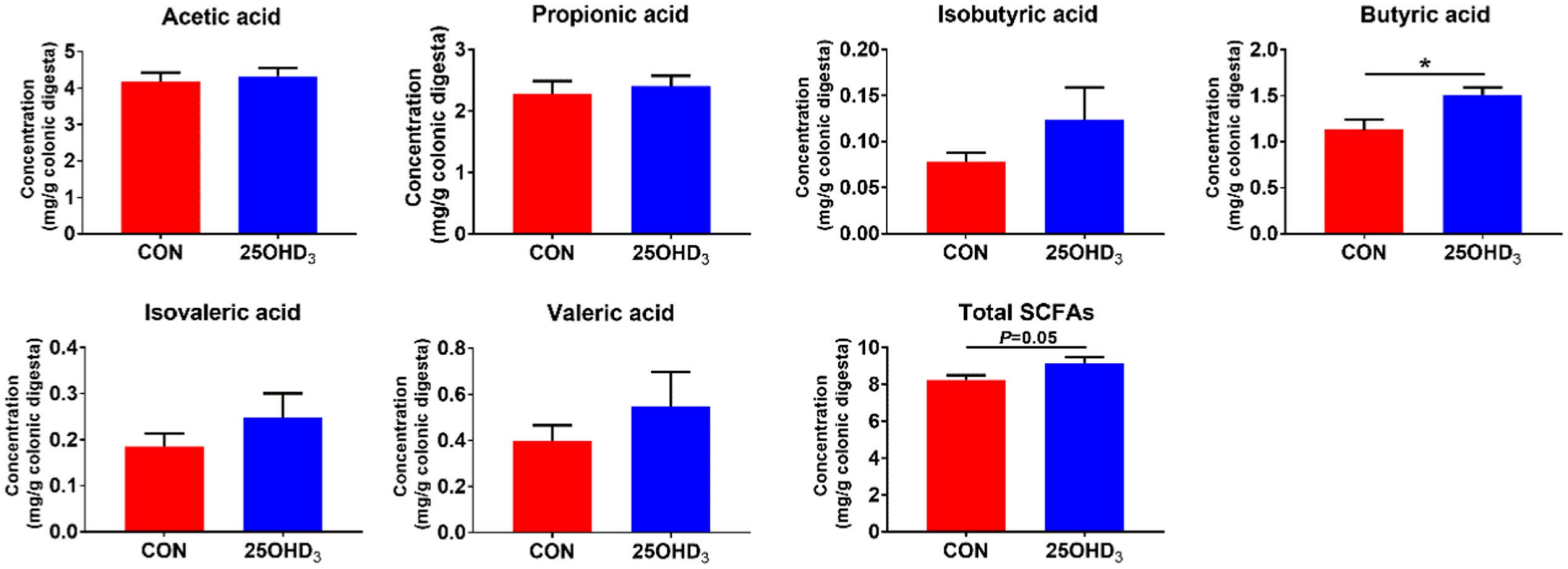
The concentrations of SCFAs in the colonic digesta. Values are shown as the means ± SEM, *n* = 5. *p < 0.05 versus CON group.

As shown in Figure 7, 25OHD_3_ enhanced (*p* < 0.05) the concentration of butyric acid and tended to increase total SCFAs in the colonic digesta compared with control.

### Correlation between the colonic abundance of *Lactobacillus* and serum parameters, pork quality parameters, bone parameters or colonic metabolites

**Figure 8:**
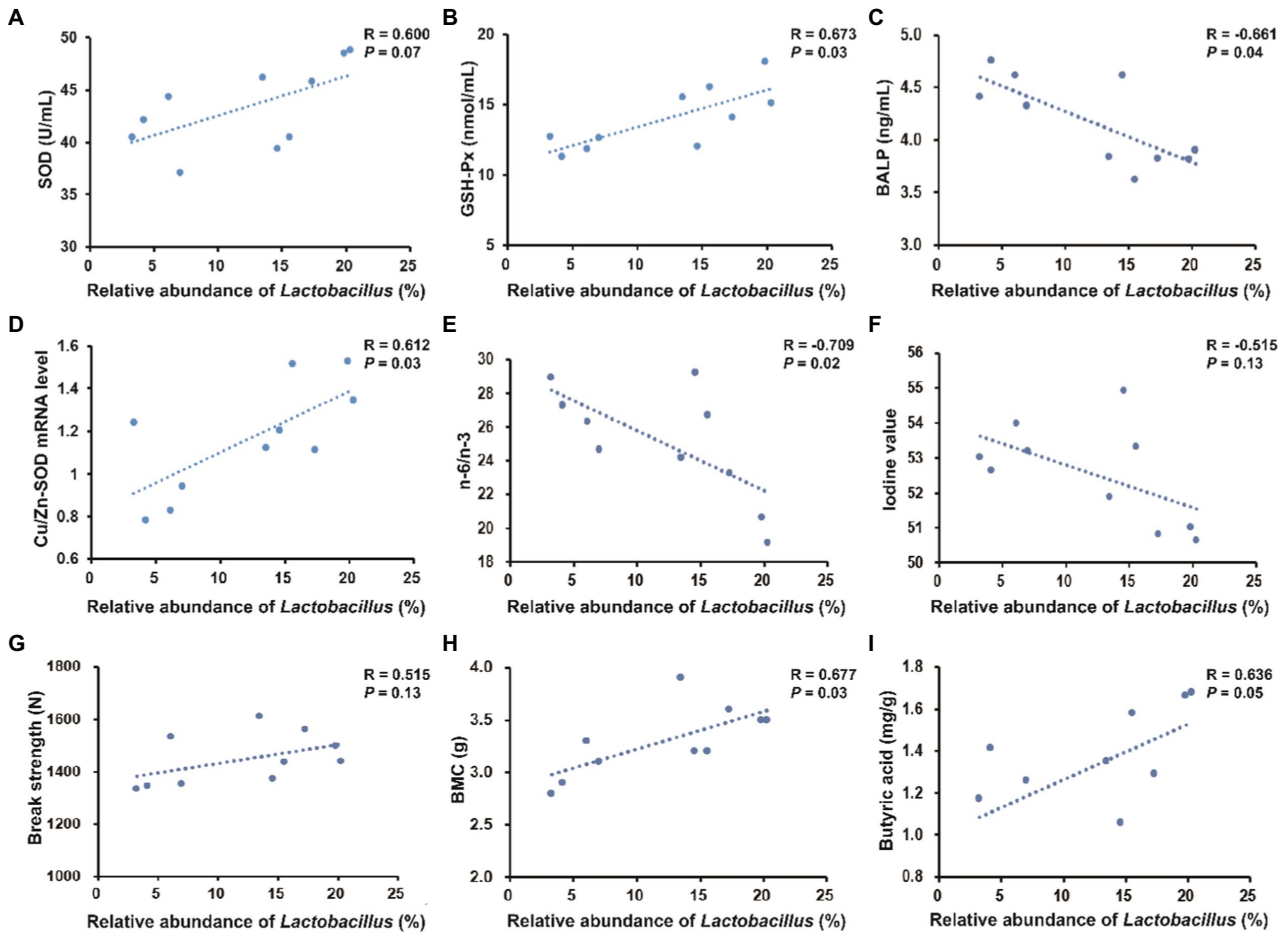
Spearman’s correlation between the relative abundance of *Lactobacillus* in the colonic digesta and serum parameters, pork quality parameters, bone parameters (take the third metacarpal bone for example) or colonic metabolites. **(A–C)** Correlation between the colonic abundance of *Lactobacillus* and serum parameters. **(D–F)** Correlation between the colonic abundance of *Lactobacillus* and pork quality parameters. **(G,H)** Correlation between the colonic abundance of *Lactobacillus* and bone parameters. **(I)** Correlation between the colonic abundance of *Lactobacillus* and butyric acid content.

As shown in Figure 8, the colonic abundance of *Lactobacillus* was positively correlated with serum GSH-Px (*R* = 0.764, *p* < 0.05) and negatively correlated with serum BALP (*R* = −0.755, *p* < 0.05). The colonic abundance of *Lactobacillus* was positively correlated with Cu/Zn-SOD expression level (*R* = 0.713, *p* < 0.05) and negatively correlated with n-6/n-3 PUFA in *longissimus dorsi* (*R* = −0.691, *p* < 0.05). The colonic abundance of *Lactobacillus* was positively correlated with BMC in the third metacarpal bone (*R* = 0.700, *p* < 0.05).

## Discussion

### Performance and carcass traits

The EFSA recommended that 50 μg/kg 25OHD_3_ is a safe dose for pigs (EFSA, 2009). Therefore, 50 μg/kg 25OHD_3_ was used in this study. Dietary 25OHD_3_ supplementation decreased the FCR of growing-finishing pigs during day 61–88 and day 1–88, suggesting that 25OHD_3_ can partly promote the performance of growing-finishing pigs fed with low *P*. Consistent with previous results reported by Zhang et al., (2021b), our study demonstrated that 25OHD_3_ supplementation did not influence carcass traits of pigs fed with low *P*.

### Pork quality and fatty acid profile

Color is a direct visual sensory attribute that is a determinant of consumer perceptions of fresh pork. Our results showed that 25OHD_3_ enhanced muscle a* value, which may be explained by the increased antioxidant ability that is related to vitamin D supplementation (Hansen et al., 2012). Moreover, 25OHD_3_ supplementation increased muscle Ca content compared to control. Supplementation of 25OHD_3_ improved the retention of Ca and P (Regassa et al., 2015), which may promote muscle contents of Ca and P and increase meat tenderness and postmortem proteolysis, so as to provide humans with high-quality meat.

For animal-derived food, fatty acid profile can affect the nutritional value and edible quality of meat. Maintaining an appropriate fatty acid profile is necessary to provide humans with high-quality pork. Higher n-6/n-3 PUFA can be involved in the pathogenesis of several diseases, including osteoporosis, inflammatory diseases, and cardiovascular disease (Hibbeln et al., 2006; Simopoulos, 2006). Higher dietary n-6/n-3 PUFA may contribute to the synthesis of inflammatory mediators and result in the pathological manifestations of metabolic syndrome. Therefore, lower n-6/n-3 PUFA in meat and derived products is important for human health. This study reported that 25OHD_3_ markedly reduced n-6/n-3 PUFA in *longissimus dorsi*, suggesting that 25OHD_3_ can improve pork quality in an important aspect related to consumer acceptability. Iodine value is a comprehensive evaluation of the unsaturation of fatty acids, which is an indicator of rancidity or the percentage of unsaturated fatty acids (Hugo and Roodt, 2007). Increased value means softer flesh, higher unsaturation and shorter shelf life. The current results showed that 25OHD_3_ decreased the iodine value of *longissimus dorsi* compared to control, indicating that 25OHD_3_ has beneficial effects on the quality of lipid profile in *longissimus dorsi*.

### Antioxidant capacity

The oxidative injury results from an imbalance between the generation of reactive oxygen species (ROS) in the body and their removal by the antioxidant systems. ROS can be scavenged by non-enzymatic components and various antioxidant enzymes such as GSH-Px, CAT and SOD (Valko et al., 2006; Wang et al., 2009). Generally, MDA level in blood and tissue is related to lipid peroxidation, reflecting the degree of damage induced by free radicals (Pirinccioglu et al., 2010). In this study, 25OHD_3_ markedly increased the serum contents of SOD and GSH-Px. Moreover, oxidative injury impairs mammalian tissue, especially the intestines (Yin et al., 2013). Our results showed that 25OHD_3_ significantly improved mucosal GSH-Px activity in the duodenum and ileum and tended to increase jejunal GSH-Px activity. The improvement of intestinal antioxidant status can alleviate mucosal oxidative injury and further decrease the impairment of the intestinal mucosal barrier. Insufficient Ca level can cause a marked reduction of antioxidant enzymes including SOD (Choi and Jung, 2017), and insufficient P level can decrease the activities of antioxidant enzymes and the relative mRNA expressions of antioxidant-related genes in young grass carp (Chen et al., 2017). Previous studies have shown that dietary 25OHD_3_ supplementation promoted intestinal absorption of Ca and P, and increased serum contents of Ca and P (Zhang et al., 2019, 2022c), which can partly explain the increase in antioxidant status of growing-finishing pigs in this study. 25OHD_3_ also reduced serum MDA level, indicating that 25OHD_3_ has beneficial effects on decreasing the degree of lipid peroxidation. For muscle antioxidant status, 25OHD_3_ supplementation prevented the increase of MDA concentration, suggesting that 25OHD_3_ has beneficial effects on reducing lipid peroxidation in *longissimus dorsi*. We also found that 25OHD_3_ tended to increase muscle contents of T-AOC and SOD. Keap1 is a negative regulator of Nrf2, sequestering Nrf2 in the cytoplasm and inhibiting its nuclear translocation (Ma, 2013). Downregulation of the Keap1 mRNA level promotes nuclear translocation of Nrf2, inducing gene expressions of the antioxidant enzymes in mice (Reisman et al., 2009). In this experiment, lower Keap1 mRNA expression and higher Nrf2 mRNA expression were observed in pigs fed with 25OHD_3_. It has been reported that Nrf2 can upregulate gene expressions that encode antioxidant enzymes, including Cu/Zn SOD and CAT (Yin et al., 2013). Our study indicated that the inclusion of 25OHD_3_ upregulated Cu/Zn SOD mRNA level. Therefore, the increased mRNA level of Cu/Zn SOD may be partly explained by higher nuclear translocation of Nrf2 caused by lower Keap1 mRNA level, further inducing an increase in muscle SOD activity.

### Bone quality

Bone markers include bone formation and resorption markers. BALP is one of the bone formation markers reflecting the activity of osteoblasts (van Straalen et al., 1991), while TRAP is one of the bone resorption markers reflecting the activity of osteoclasts (Minkin, 1982). A previous study showed that rats fed with low dietary Ca had increased alkaline phosphatase (ALP) in plasma and bone, but they lost bone Ca content (Tanimoto et al., 1991). Compared with control, low Ca concentration in the medium resulted in increased ALP activity in bone cells and extracellular matrix (Yoshimura et al., 1996). The previous results suggested that insufficient Ca improved the activity of osteoblasts to produce more ALP, and higher ALP activity may be a compensatory mechanism in low Ca state. The present study reported that 25OHD_3_ markedly decreased serum BALP concentration, indicating that the inclusion of 25OHD_3_ in low P diet may decrease the utilization of Ca and P in bones and increase the utilization of Ca and P in diets.

Insufficient Ca level can promote the generation of PTH, inducing the release of bone minerals, while imbalanced P metabolism can affect bone integrity and strength. The present study demonstrated that supplementation of 25OHD_3_ markedly enhanced bone Ca content and BMD in the metacarpal bone of pigs, indicating the potential influences of 25OHD_3_ in low P diet on increasing bone mineralization. It has been reported that higher mRNA expressions of vitamin D receptor and calcitropic genes triggered by 25OHD_3_ supplementation would be the potential mechanism underlying the positive effects of 25OHD_3_ on calcium deposit and bone quality (Zhang et al., 2019). For bone mechanical properties, strength and stiffness are important parameters to evaluate the biomechanical quality of the entire bones. Among mechanical parameters, strength refers to the maximum load that a structure can withstand before failure, and is associated with bone fracture and injury (Venkatesan et al., 2015). Stiffness is the slope of linear part of the load–displacement curve, which represents the ability of the structure to resist deformation under load (Zhang et al., 2019). The present research demonstrated that the 25OHD_3_ improved strength and stiffness, indicating that the inclusion of 25OHD_3_ in low P diet effectively improved the biomechanical property of the porcine metacarpal bones.

### Colonic microbiota

The inclusion of Ca and P in diets play an important role in modulating the diversity and composition of gut bacteria and influencing the gut health of pigs (Zhang et al., 2022c). It has been reported that free Ca suppresses the proliferation of specific bacteria in the proximal gut (Larsen et al., 2007; Metzler-Zebeli et al., 2010), while P acts not only on commensal bacteria, but also on pathogenic bacteria (Heyer et al., 2015). Lower Ca and P levels in rat diets decreased *Lactobacillus* abundance in the ileum and feces and increased the amount of *Salmonella enteritidis* (Ten Bruggencate et al., 2004), suggesting that Ca and P deficiency in diets can hamper intestinal barrier function. Moreover, insufficient P can reduce bacterial cellulose fermentation, in turn decreasing SCFA production (Heyer et al., 2015). In general, increased α-diversity has beneficial effects on maintaining immune homeostasis in the gut (Ciocan et al., 2018). Our study indicated that 25OHD_3_ supplementation enhanced a-diversity in the colonic bacteria, as indicated by a higher Chao 1 index, which may help maintain intestinal immune homeostasis. Firmicutes and Bacteroidetes were the major phyla in the colonic digesta, which is similar to the data described by Zhang and Piao (2022a). Down to the family level, 25OHD_3_ increased the colonic abundances of Lactobacillaceae and Bacteroidales_S24_7_group compared with CON group. Lactobacillaceae is considered to be beneficial bacteria that modulates gut health *via* increasing mucosal barrier integrity and immunity (Zhang et al., 2020). Bacteroidales_S24_7_group is regarded as a butyrate-producing bacteria with beneficial effects on intestinal function and health (Villanueva-Millán et al., 2015). Our results showed that the inclusion of 25OHD_3_ increased colonic butyric acid content compared to control, which was in agreement with higher abundance of Bacteroidales_S24_7_group. Butyric acid has several beneficial functions, such as proliferation and differentiation of gastrointestinal epithelium, alleviation of inflammatory responses, reinforcement of barrier function and modulation of intestinal microbiota (Bedford and Gong, 2018). At the genus level, colonic *Lactobacillus* abundance was higher in the 25OHD_3_ group than that in control. *Lactobacillus* is taken as a potentially beneficial bacteria, which can inhibit the colonization or infection of pathogenic bacteria by competing for nutrients and epithelial binding sites and the growth of pathogenic bacteria by producing active ingredients such as bacteriocins and lactic acid (Kailasapathy and Chin, 2000). As a result, the improved gut bacterial community and butyrate production may partly contribute to promoting intestinal function and growth performance in pigs fed with 25OHD_3_ supplementation. Collectively, our study suggested that 25OHD_3_ supplementation in low P diet improved intestinal function by regulating bacterial diversity and composition and increasing the production of their metabolites.

The present study showed that the colonic abundance of *Lactobacillus* was positively correlated with serum SOD activity and significantly positively correlated with serum GSH-Px activity. Thus, improved antioxidant activity in pigs fed with 25OHD_3_ may be associated with gut microbial changes. The gut microbiota-muscle axis is important in modulating muscle growth and development *via* beneficial or harmful microbial metabolites (Bindels and Delzenne, 2013). A previous study suggested that a probiotic, *Lactobacillus johnsonii* can significantly prevent *Clostridium perfringens* infection-induced increase in n-6/n-3 PUFA in the breast muscle of chickens (Wang et al., 2017). In this study, the colonic abundance of *Lactobacillus* was significantly positively correlated with Cu/Zn-SOD expression level and negatively correlated with n-6/n-3 PUFA in *longissimus dorsi*, indicating that the increased abundance of *Lactobacillus* was potentially related to the improved pork quality by 25OHD_3_ supplementation. However, the direct contribution of *Lactobacillus* to pork quality is still unclear and deserves further study. In addition, gut microbiota can regulate bone mass in mice (Sjögren et al., 2012) and the preferential bacterial genus that has beneficial effects on bone development is *Lactobacillus* (Villa et al., 2017). *Lactobacillus* can modulate mineral metabolisms such as an increase in calcium solubility and absorption due to the production of short-chain fatty acids (Parvaneh et al., 2014). In this study, the correlation analysis showed that colonic *Lactobacillus* abundance was significantly negatively correlated with serum BALP and positively correlated with BMC in the third metacarpal bone. This result confirmed that the increased *Lactobacillus* abundance may be associated with improved bone quality in pigs fed low P diet with 25OHD_3_ in our study, but the underlying mechanisms involved need to be further investigated.

## Conclusion

In summary, the inclusion of 50 μg/kg 25OHD_3_ in low P diet partly improved production performance, pork quality, antioxidant status, and bone properties of growing-finishing pigs. Moreover, supplementation of 25OHD_3_ in low P diet increased the colonic abundance of *Lactobacillus*, which was positively correlated with serum antioxidant status, pork quality, and bone characteristics. This study provides a new perspective for improving pig performance with reduced use of exogenous P in diets.

## Data availability statement

The data presented in the study are deposited in the NCBI repository, accession number PRJNA914891.

## Ethics statement

The animal study was reviewed and approved by The Institutional Animal Care and Use Committee of China Agricultural University.

## Author contributions

LZ: investigation, data curation, formal analysis, writing-original draft, conceptualization, methodology, software, and writing-review and editing. SL: investigation. HW: conceptualization, methodology, writing-review and editing, and supervision. XP: conceptualization, methodology, writing-review and editing, supervision, and funding acquisition. All authors contributed to the article and approved the submitted version.

## Funding

This research was financially supported by the National Natural Science Foundation of China (31772612 and 32202725), the Beijing Municipal Natural Science Foundation (6202019), and China Postdoctoral Science Foundation (2022M723370).

## Conflict of interest

The authors declare that the research was conducted in the absence of any commercial or financial relationships that could be construed as a potential conflict of interest.

## Publisher’s note

All claims expressed in this article are solely those of the authors and do not necessarily represent those of their affiliated organizations, or those of the publisher, the editors and the reviewers. Any product that may be evaluated in this article, or claim that may be made by its manufacturer, is not guaranteed or endorsed by the publisher.

## Footnotes

^1^ http://drive5.com/uparse/

^2^ http://rdp.cme.msu.edu/

^3^ https://cloud.majorbio.com/
